# From the laboratory to the consumer: Innovation, supply chain, and adoption with applications to natural resources

**DOI:** 10.1073/pnas.2115880119

**Published:** 2022-06-01

**Authors:** David Zilberman, Thomas Reardon, Jed Silver, Liang Lu, Amir Heiman

**Affiliations:** ^a^Department of Agricultural and Resource Economics, University of California, Berkeley, CA 94720;; ^b^Department of Agricultural, Food, and Resource Economics, Michigan State University, MI 48824;; ^c^Department of Agricultural Economics and Rural Sociology, University of Idaho, ID 83844;; ^d^Department of Environmental Economics and Management, Hebrew University of Jerusalem, Rehovot 7610001, Israel

**Keywords:** adoption, supply chain, marketing, innovation, policy

## Abstract

We analyze the transition from innovative ideas to final marketed products. This transition occurs through two synergetic supply chains for innovation and products. Basic concepts are developed, tested, upscaled, and introduced to commercial use in the innovation supply chain. Then, the products are produced and delivered to the consumer through the product supply chain. We argue that product markets trace their birth to product innovations. These markets tend to start as noncompetitive, which rewards innovators. Credit access and risk determine the reliance on contracting and product diffusion over space and time. The innovation and product supply chains are encouraged and facilitated by public policies, such as support for research and education, intellectual property rights protection, low barriers to trade, science-based regulation, and well-functioning capital markets. We argue for multidisciplinary research incorporating knowledge from economics, business, and engineering to better understand the evolution of innovative companies and supply chains. This understanding will help the development of policies to address challenges of climate change and food security among others.

## Significance

Research on innovation has two strands: institutions undertaking innovation as a research and development process and companies commercializing innovative products. We combine these strands, analyzing a sequence going from an innovation supply chain to a product supply chain from laboratory to market. We argue that these supply chains are symbiotic, and the relationship between entities is affected by economic considerations. Our framework allows an understanding of how research, regulatory policies, and economic conditions affect the emergence of innovations, the creation of institutions (markets, firms, contracts) to carry out these innovations, and the diffusion of the resulting products. Our approach may improve the design of strategies to induce climate change and food security solutions.

Policy makers, scientists, and the public are interested in understanding the processes that lead to the transition of ideas to new products and services. We present a framework to analyze this transition consisting of two symbiotic supply chains: the innovation supply chain (ISC) and the product supply chain (PSC). In the ISC, scientific discoveries are made, developed, and adapted into marketable products. In the PSC, the innovating firm forms expectations of the demand for their products, designs a supply chain to produce and market its products, and then, implements its supply chain while adapting it as reality unfolds

The behavior of these supply chains is not mechanical. Instead, the organizations that manage the different supply chain segments are motivated by economic and political incentives. For example, a firm introducing a new fruit will use profitability considerations to decide how much to market and to what extent to produce the food itself vs. relying on contractors. Supply chains adapt to shocks. For example, the COVID-19 pandemic led ISCs to develop vaccines and PSCs to distribute the vaccines. The pandemic also led to the expansion of food delivery from retailers to consumers. Furthermore, the relationship between the ISC and PSC is symbiotic and synergetic, with multiple feedbacks. Consumer feedback obtained through the PSC may lead to investments in research to improve product quality, and new product properties discovered in the ISC will lead to a modification of the PSC. Our analysis extends, relies on, and complements economic frameworks for analyzing, innovation, diffusion, and industrial organization.

Our framework applied to innovations in general, spanning developed and developing countries. Innovations are transformative to varying degrees. Most are incremental; some are radical, but all need supply chains. Our framework is quite general, but its application may vary according to circumstance. For example, the supply chain design may be more challenging for a transformative innovation (such as the cell phone) than for an incremental innovation that relies upon and modifies existing supply chains (such as a new variety of beer). The analysis of the ISC and PSC applies to a large extent for most sectors in high-income countries, for fewer in middle-income countries, and for few sectors in low-income countries.

The paper includes five sections.

1) We analyze the ISC, emphasizing the division of labor between the public and private sectors and the role of public funding of research that provides public goods.
2) We review the PSC. We present a framework for projecting adoption and thus, demand over time. It emphasizes the heterogeneity between consumers and the vital role of learning and marketing in shaping adoption dynamics. Then, we introduce a framework to analyze the dynamics of the PSC. We emphasize that the design of a supply chain is an economic problem. The innovating firm designs and implements a supply chain to procure inputs, produce, and market the innovative product or technology. For example, a firm introducing a new fruit needs to decide how much, how to market, and the extent of reliance on contractors for production. The analysis emphasizes that markets, firms, and products are the endogenous outcomes of innovation and PSCs and suggests that supply and demand are interdependent, with firms engaging in marketing activities to generate demand.
3) We analyze the symbiotic relationship between the ISC and PSC and identify feedback loops between the different stages of the two. We suggest that the features of both supply chains depend on polices market conditions using illustrative examples. We argue that government regulations of markets must balance the incentive for innovation with the consequences of excessive market power.
4) We present implications for environmental, natural resources, and food management policies, arguing that shocks, like climate change or pandemics, affect firms directly and through their impacts on other economic agents throughout their supply chains. Thus, more attention to supply chain design and function will improve efforts to mitigate climate change and address food security and health challenges.
5) The conclusion section identifies directions for future research. It emphasizes the need for collecting data and empirical analysis on supply chains evolutions. It highlights the importance of studying technological change within the context of multistage supply chains, relying on findings from economics, engineering, and other disciplines.

## The ISC

Innovation is doing things in a new way. Innovation can include new products, technologies, services, institutions, and policies. In its modern form, the ISC is referred to as the educational–industrial complex. The “upstream” of the ISC is the supplier of “basic science,” which can be a laboratory in a public institution or a private firm. The past two centuries have seen a shift from individual or “small science” teams to large teams and laboratories—“big science” (1). The outcomes of basic science are uncertain. Much of the findings are public goods available to all members of society. Basic research also identifies side effects (externalities) of economic activities (climate change, loss of biodiversity), and activities to control them that are more valuable to society than to individual firms. To a large extent, the public sector finances the basic research since the returns on investment in most basic research projects are much higher from a social perspective than a business perspective. Studies have found that based on social rates of return, there tends to be underinvestment in public research (2).

In the midstream of the ISC, innovative ideas are transformed to inventions, upscaled, and tested for efficacy and impacts by start-ups, firms, and public sector laboratories. Some of the basic research discoveries have implications that can lead to commercially viable innovations (including both tangible technologies and intangible processes) and the rights to use public sector innovations conferred to the private sector by offices of technology transfer (OTTs). The OTTs sell the right to use their patents to companies and assist in their utilization. Patent rights serve as an incentive for investment in product development (3). While universities emphasize basic research, the private sector invests mainly in applied research, so basic research and applied research tend to complement, not substitute. Frequently, university scientists participate in implementing their innovation and are partners in firms. For example, Boyer and Cohen discovered recombinant DNA technology as researchers at University of California, San Francisco and Stanford, which jointly held the patent. Boyer relied on this patent as the critical element when he cofounded Genentech (4).

Most innovations tend to build on existing technologies or processes. Incremental innovations may be introduced and developed within laboratories and by practitioners in companies. Innovators recombine existing innovations and concepts from different fields to generate new products. Major inventors, like Edison and Ford, relied on existing technologies to develop new products. Product design companies, like IDEO in Palo Alto, specialize in designing products or processes for clients (5).

The downstream of the ISC establishes the processes for the product's commercial production. This industrial design requires combining technical knowledge with expectations of demand and input prices.* Large firms frequently conduct this process after acquiring start-ups that initially upscaled the innovation, frequently in partnership with marketing and industrial design firms.

Governments support the ISC directly through support of research. Investment in research is affected by scientific, economic, security, and political considerations. Global expenditure on research and develoment has quadrupled between 1980 and 2016, reaching close to $2 trillion in 2016. The share of the United States in global research and develoment has declined to less than 30%, while the share of Asia has been increasing. The share of private sector research in total research and develoment expenditures has increased to become close to 70%. Private research in agriculture in developed and middle-income countries is close to 60%. It is less than 25% in low-income countries, where donors augment local governments in supporting public research (7).

Governments also support the ISC through support for education, and through establishing mechanisms to protect intellectual property. Gilbert and Shapiro (8) investigated the optimal design of patents, emphasizing the trade-off between incentivizing innovation and limiting abuse of market power. Society has realized that implementing innovation may require extra risk and therefore, grants patents and monopoly power, but the market power held by innovators can be abused, for example, by blocking new entrants. The outcomes of the ISC are the foundations for much of the PSC.

## PSC

The supply chains for incremental innovations may modify existing systems, but new supply chains will be built for radical innovations. The innovating firm undertakes several activities to establish a PSC. First, the firm estimates diffusion patterns to select the most promising specification of the innovation. Second, it designs a supply chain and marketing program. Third, it implements the plans. The transition from the ISC to the PSC may not be distinct. Firms may consider various combinations of products, production systems, and supply chains while assessing their profitability. Research on adoption and supply chain design provides important tools to guide the establishment of a PSC.

### Assessment of Potential Adoption and Diffusion of the Innovation.

First, the firm selects a framework to analyze the potential diffusion of its innovation. Adoption of an innovation can be measured by either discrete variables (such adopting a tractor or not) or continuous variables (share of land planted with a new variety). Adopted innovations may have one component or a package of components: for example, hardware and software of a computer. Diffusion is aggregate adoption and is measured by the share of potential adopters that adopt the product or technology at a given moment or the share of productive capacity utilizing new technology (9).

Diffusion has been analyzed with various models. The first is the Rogers (10) imitation model, where diffusion tends to be an S-shaped function of time:

[1] $$P_{t}=\frac{k}{\left(1+e^{-\left(a+bt\right)}\right)},$$

where $P_{t}$ is the fraction of the population that had adopted the product or technology up to time *t*, k≤1 is the diffusion rate once the product or technology is fully adopted, the parameter *a* is a measure of initial adoption, and *b* parameterizes the rate of diffusion. Griliches (11) developed an econometric framework to estimate the parameters of this model, showing that they are affected inter alia by profitability and other economic considerations. Griliches’ framework has evolved into the Bass diffusion model that has been applied in the marketing and management literature (12).

The S-shaped diffusion curve of the imitation model is borrowed from epidemiological models of the spread of a disease. The beginning of the diffusion process features a low but increasing adoption rate, with the adoption rate rising through the “bandwagon effect,” in which peers follow adopters. Eventually, the adoption rate reaches its peak, begins to slow, and finally, stops as greater aggregate adoption depletes the number of potential adopters. Recent literature has expanded the imitation model to include communication across electronic social networks (13).

Imitation models do not have an explicit economic mechanism of decision-making, but they are effective frameworks to estimate diffusion. An alternative model of diffusion, the threshold model, was introduced by David (14) and extended and formalized by Zilberman et al. (15). The threshold model assumes that diffusion is determined by three elements: individual decision-making, heterogeneity, and dynamics.

Individual decision-making (10) includes several stages: awareness, evaluation, decision, and reevaluation. Potential adopters may be aware of the product or technology through formal means, such as the media, salespersons, or extensions, or informal sources, such as word of mouth and observing others’ choices. The threshold approach recognizes the role of social networks and opinion leaders to adoption through their impacts on awareness and assessments needed for evaluation, as in Valente and Davis (16). It assumes that heterogeneous end users use different decision criteria that include profits over time, risk, and discounting and face differential constraints on resources, credit, and decision-making capacity. Because of heterogeneity, the timing of adoption varies, and good marketing strategies aim to identify the “low-hanging fruit” first.

Furthermore, dynamic processes are likely to increase the range of adopters over time and propel diffusion. These include learning from the experience of adopters, learning by using (improved utilization of the technology by adopters), learning by doing (reducing the costs of supplying the new technologies), and network externalities (increased value of a technology, like telephones, as the number of adopters increases).

Second, the firm undertakes an analysis of actual and potential adoption. The applications of the imitation and threshold models can be complementary. The firm may use the threshold model in designing policies and strategies for launching and managing the commercialization of innovations and in integrating marketing mechanisms that can induce adoption. The imitation model has a relative advantage in assessing awareness of an innovation and can be used to estimate diffusion rates.

Econometric and statistical analyses aim to identify some of the key elements of the modified threshold model and, in particular, to assess the decision criteria of different potential adopters: to what extent they are affected by risk, are constrained by credit, and deviate from economic rationality. Furthermore, the economic literature aims to identify sources of heterogeneity among potential buyers, as it affects the adoption of specific technologies (17) as well as some of the parameters of the dynamic processes, like learning by doing.

On the other hand, innovating firms have ex ante analytical tools. Experimentation is key in developing new technologies and institutions. Innovating firms may engage in pilot studies before they fully embark on new technology. They may do demonstrations to potential end users to assess demand and adjust the product attributes (18).

Economists have recognized the value of using experiments to assess the viability of new technologies and practices (19) and their likelihood of adoption (20). The notion of experimenting before taking action also applies to institutional innovation. The Chinese government experimented with using market mechanisms to allocate resources in one region before introducing nationwide reforms (21).

Marketing analysts use a broad range of prelaunch predictive tools that are based on finding out the importance of the innovation, the willingness to consider it, the importance of specific attributes and consumers’ willingness to trade off these attributes, and the willingness to pay for the innovations in different forms (22). Marketing research tools, such as conjoint analysis, are widely used to estimate the importance of the different attributes and price and then design the new product policy (23). Partial least square models have been combined with structural equation modeling to incorporate causality in predictions (24).

Diffusion patterns are the outcome of choices by both the adopters (who form the demand) and suppliers of technologies. The sellers need to quantitatively assess adoption behavior and how their decisions affect it. These decisions include pricing, selection of marketing tools, and location of supply centers and stores. Firms will make these choices by maximizing expected discounted profits consisting of revenues minus marketing and supply costs. In their design of the optimal strategy, firms adopt the commonly used segmentation, targeting, and positioning principle, which starts by identifying segments, targeting (choosing the right segments and prioritizing the segments), and positioning (matching the product to the specific characteristic of the chosen segment) (25, 26). The introduction of improved marketing tools (e.g., segmentation followed by targeting information through social networks) may modify and accelerate the diffusion of new technologies (27).

The literature on adoption and diffusion identifies constraints to adoption (risk, ability, lack capacity, regulation) and emphasizes that “attribute bundling,” whereby producers tailor their product to address customer constraints, can enhance adoption. Because early adoption generates positive informational externalities, as it leads to learning by both suppliers and potential adopters, there is a case for subsidization of early adoption.

Traditional expected utility models have shown that attitudes toward risk affect adoption. A high degree of risk aversion may reduce the likelihood, speed, and scale of adoption of risky innovations (where risk is defined as a larger variability of returns around the average). Recent examples from agricultural technology adoption include Karlan et al. (28), Emerick et al. (29), and Donovan (30) among others. Prospect theory introduces concepts such as loss aversion and overweighting of small probabilities that may similarly reduce technology adoption (31). Moreover, adoption of new and innovative technologies frequently requires high fixed costs in terms of monetary investment and effort. In deciding when to adopt, end users weigh the benefits of immediate adoption against the option to delay, which saves interest costs, may lower fixed costs, and lowers risk as more information about the technology becomes available (32). Potential adoption of technology may be affected both by lack of knowledge about the performance of the technology as well by “fit risk,” the extent to which the technology fits the consumer’s needs and capabilities (33).

The innovating firm accounts for these risk considerations when estimating how the innovation’s risk profile will affect adoption and what “bundling” of services might be needed to encourage adoption. For example, the innovator firm may bundle an insurance mechanism with the product, such as “money-back guarantees” and warranties and/or allowing potential adopters to try the innovation for a limited time by offering product demonstrations (34). Prepurchase experimentation allows potential buyers with “low fit” to forgo adoption, and return policies reduce the costs of poor fit for the end user, reducing adoption risks (33).

Moreover, the innovating firm’s communication (including advertising) to potential end users informs the latter’s perception of the innovation’s risk and utility and can reduce information asymmetry. Innovating firms can employ various marketing tools, such as advertising, branding, product demonstrations, product samples, and warranties, to reduce various risks, as emphasized in the marketing literature. For example, the innovating firm can develop a brand that conveys high quality and low risk to a potential consumer (33). Moreover, the potential end users of an innovation may face concerns over access to complementary products, inputs, or spare parts for it. Firms mitigate this risk in many ways. Manufacturers of equipment have local dealers that provide customers with parts and repairs. Computer firms provide access to software and training. The innovating firm may also provide needed complementary inputs to its suppliers; for example, the supermarket chain Carrefour works with banks and seed companies to provide farmers access to seeds and credit to grow differentiated crop varieties in Indonesia (35).

The capacity of potential adopters affects diffusion. As the threshold model suggests, potential adopters are heterogeneous in their resource availability (farm size, wealth, income), demographics, and biophysical conditions. There is ample evidence that larger farmers are likely to be early adopters of indivisible technologies, like tractors and computers (2, 36, 37).

Differences in allocative ability (38) may lead individuals to follow different decision rules, where some are early adopters and others are followers. Wuepper and Lybbert (39) review evidence finding that early adopters tend to be confident in their ability to control the effects of technologies they adopt.

The innovating firms sometimes adapt the product to the capacity and needs of the adopter.

For example, they may supply a smaller cheaper version of the product to smaller farms or firms, as occurs in the farm machinery sector. They also sometimes “bundle” alternative payment schemes, such as leasing and credit, to enable adopters to overcome credit constraints and transaction costs. The innovating firm recognizes that adoption of the innovation can affect the adopter’s valuation of its assets, such as when irrigation increases the value of irrigated land (40), and this change in valuation can ease credit constraints.

Institutions and policies affect risk (e.g., through public insurance schemes), transaction costs (e.g., through public investment in infrastructure), and credit (e.g., through micro- and cooperative credit arrangements). While scale limitations may prevent individuals from purchasing a new product, technology suppliers may introduce rental or custom service (41, 42). Emerick and Dar (43) showed that a short field school overcame information limitations and led to the adoption of flood-tolerant rice in India.

Governments can also enact policies that create an incentive for adoption, such as emission taxes or technology standards that favor a pollution-reducing technology (44) and renewable fuel standards to enhance the use of biofuels in the United States (45). In extreme cases, governments can force the adoption of new technology when they ban the usage of the existing technology (the United Kingdom plans to ban diesel and gasoline cars in 2030 to encourage electric vehicle adoption). Consequently, innovating firms may engage in lobbying for policies that enhance adoption (46).

### Design and Implementation of Supply Chains of Innovated Products/Technologies.

Once firms have formed their expectations for adoption patterns and demand, they can proceed with designing a supply chain. We present a conceptual framework for the design and management of supply chains to implement innovations. Supply chain design has not been emphasized in economics. Economic theory was formed when agriculture was the dominant industry, and therefore, perfect competition was a natural benchmark. While traditional economies were in a state of static equilibrium, modern economies change through innovation and accumulation of human capital and are in constant disequilibrium (38). Schumpeter (47) emphasized the crucial role of innovation and creative destruction. Coase (48) and Williamson (49) emphasized that the firm producing a product may engage in a sequence of multiple activities and decisions about how much to produce inside the firm vs. outside the firm, which depends on economic and institutional conditions. Our framework is complementary to macroeconomic frameworks of Aghion et al. (50) and Acemoglu (51), who documented the role of innovation, institutions, and structural change, in inducing economic growth.

Our analysis is based on the assumption that implementation of innovation is through supply chains with multiple stages. While we present evidence that supports this assumption, it should be further investigated. Here, we present a basic dynamic model to obtain decision rules under certainty. We further suggest incorporating more complex aspects of reality into the analysis (e.g., risk, credit, competitors), mainly relying on the literature. We assume that an entrepreneur has an innovation that is the output of the ISC. The key questions facing the entrepreneur are the scale of operation and the supply chain structure: in particular, the extent and manner of using external suppliers of inputs (35, 52). One contribution of our analysis is that we suggest that innovation leads to changes in the structure of multiple markets throughout the supply chain. While Sutton (53) and others consider strategic interaction among firms and emphasize how innovations modify final product markets, we highlight the impact of the innovation on intermediate input markets.

To describe the innovator firm’s choices in the design of its supply chains, we introduce a simple two-stage dynamic supply design chain model, building on the static framework of Du et al. (54). We assume that the firm is producing a new differentiated product (such as electric cars) and may use differentiated intermediary inputs (such as batteries). Thus, in the model, the innovative firm is likely to have market power in both input and output markets. This microeconomic model is unique because it allocates resources between the upstream and downstream of the supply chain and determines the share of reliance on external and internal inputs within a dynamic setup. For simplicity, we assume that each unit of output at period *t*, $X_{t}$, is produced by one unit of intermediary input (e.g., corn for biofuel). Thus, total intermediate input quantity is $X_{t}=X_{I}{}_{t}+X_{E}{}_{t},$ where $X_{I}{}_{t}$ is inputs produced internally, $X_{E}{}_{t}$ is purchased inputs, and *t* = 0 to *t* = *T*. The entrepreneur uses estimates of the demand for the final product to estimate expected revenues for period *t*, $REV_{t}\left(X_{t}\right)$, which is a function of output. Costs are divided into the costs of intermediate inputs as well as costs of processing. The cost of inputs produced internally is $\mathrm{INFC}\;\left(X_{I}{}_{t}\right)$, and the cost of purchased inputs is $\mathrm{EXPC}\;(X_{E}{}_{t})$. The cost of processing is $\mathrm{PROC}\left(X_{t},K_{t}\right)$, and it depends on the total intermediate input and stock of capital good used in processing at time *t*, denoted by $K_{t}.$ The capital stock is augmented by investment, $I_{t}$, and depreciates at rate δ; the firm faces interest rate r. The capital accumulation equation is

[2] $$K_{t+1}=I_{t}-\delta K_{t}.$$

The objective of the firm is to maximize net present value of profit over time:

[3] $$\begin{array}{c} \mathrm{Max}\\ X_{It},X_{Et},I_{t} \end{array}\;\overset{T}{\underset{t=0}{\sum }}\frac{1}{{\left(1+r\right)}^{t}}REV_{t}\left(X_{t}\right)-\;\mathrm{PRO}C_{t}\left(X_{t},{\text{K}}_{t}\right)-(\mathrm{INF}C_{t}\;\left(X_{It}\right)+\mathrm{EXP}C_{t}\;\left(X_{Et}\right))-I_{t}$$

subject to the capital accumulation equation and nonnegativity constraints. Each period, the firm decides the number of inputs it produces and buys and the investment amount. These decisions determine the capital stock, output, revenue, costs, and profits. Profit in each period is revenue minus

• variable costs of processing,
• investment costs,
• costs of producing inputs internally, and
• costs of purchasing inputs from others.

Allocation of the sourcing of inputs in house vs. external suppliers is a function of the relative costs of each source adjusted for market power.

In particular, if the firm has a relative advantage in input production at every scale, it will be vertically integrated and produce all its intermediate inputs internally. In this case, the optimal output level is at the point where the marginal revenue per unit of input is equal to the sum of the marginal processing cost plus marginal internal production costs.^†^

Suppose the firm or external source does not have a clear advantage in producing inputs. In that case, the firm will make some of the inputs internally and buy the rest until the marginal cost of internal production equals the marginal cost of procurement.^‡^

If the firm does not have an advantage in producing its inputs at any scale, it will buy them. The optimal level will be where the marginal revenue per unit of input equals the marginal processing cost plus marginal purchasing cost.^§^

The level of investment at each period will equate the marginal discounted net present value of capital $\left(\mathrm{VMB}I_{t}\right)$ to its price. The marginal benefit from investment is the sum of the future discounted benefits of the capital it adds.^¶^ Therefore, higher depreciation and discount rates will lead to less investment.

Our analysis suggests that the innovative firm will benefit from market power in both output and input markets. For example, through (incremental) innovations, Apple created the iPhone, has market power in smartphones, and charges a relatively high price. Apple has long outsourced the production of the iPhone components and has had sufficient market power in purchasing components to keep its costs low and profits high.

The model allows the revenue and cost functions to change over time due to dynamic processes of diffusion and learning. If potential revenues increase over time and costs of processing decline, production will increase. If the costs of external input suppliers decline faster than those of the innovating firm, the share of purchased input will increase. For example, Kenya has become the largest exporter of flowers to Europe. In the late 1960s, Dansk Chrysanthemum Kultur (DCK) established a vertically integrated operation producing flowers in Kenya and shipping them. Over time, DCK began outsourcing to producers as other shippers emerged in Kenya. The Kenyan industry has since automated and digitized much of production, logistics, and marketing (55, 56). Conversely, Amazon has begun vertically integrating its delivery operations after initially relying on contractors.

Our simple model abstracts away from important considerations. We emphasize endogeneity in the input market, where the extent to which the firm makes rather than buys intermediate inputs may change over time. However, we assumed that the firm is buying in a market, while firms may issue contracts of different types to provide differentiated intermediary inputs. They may use bidding or tournament to select suppliers (56, 57). Our analysis of behavior at the output market fits cases when the innovative firm is a monopolist or is part of a monopolistic competitive structure (where multiple firms have monopoly power in markets for closely related products) and takes other firms’ behavior as given. Advanced models of industrial organization (53, 58) address strategic behavior and interaction among firms over time. Their analysis has multiple implications for our problem. First, while the innovator may start as a monopoly, over time the market structure may become oligopolistic or monopolistically competitive. Companies competing with the original innovator may have differentiated products but with a significant degree of substitutability with the original firm’s innovation. For example, Monsanto initially had a monopoly in the genetically modified corn market, but now, the market is oligopolistic. The iPhone was followed by other smartphones.

Second, the innovator may expect loss of profit and market power by new entrants and engage in defensive strategies, such as predatory pricing and acquisition of potential competitors and intellectual property. For example, to extend its market power in the seed industry, Monsanto acquired several seed companies to become the largest in the world (59). Monsanto builds its intellectual property by acquiring start-ups like Calgene. Finally, Monsanto was taken over by Bayer, a major player in life science and agriculture. Antitrust authorities approved these actions, but further research should assess their ex post impacts.

Third, continuous research and develoment leading to incremental innovations (beyond the original innovation) reduces the cost of production and improves quality. The innovations allow supply increases and price reductions and thus, enhance adoption.^#^ Plant-based meat is an example of a product where innovations reduced cost and improved the taste and appearance, leading to increased adoption and thus, market size.

Furthermore, improving quality may lead some early adopters to replace older models of the product with recent models, creating secondhand markets for outdated versions. For example, outdated tractor models are sold from China to Burmese farmers and used cars from Europe are exported to Africa. Dynamic supply chain design allows upgrades that increases profits from later versions.

Our model does not consider entrepreneurs’ attitudes toward risk. However, the reliability of the volume, timing, and intermediary input quality may be uncertain. Risk aversion will lead to relying less on riskier external suppliers and producing less total output. Similarly, riskier processing of the intermediary input is likely to lower production (42). Over time, learning and adaptation may reduce the risk of supply and processing activities and increase overall production and reliance on external suppliers.

Our basic model also ignores that, in practice, entrepreneurs operate under credit constraints, which are more restrictive in developing countries and reflect asymmetric information between borrowers and lenders (60). A credit-constrained firm that aims to expand its output capacity may use its credit to invest in increasing its output processing capacity and mostly rely on externally purchased intermediary inputs. To overcome credit costs and constraints, Tyson, the largest poultry processor in the United States, elected to continuously invest in output processing and marketing while contracting with farmers to produce its intermediate inputs (chickens). Bruce Church Farms was a large lettuce producer that introduced packaged precut salads. It started the processing company Fresh Express and sold the farm to finance a large processing plant while contracting with farmers to grow the lettuce (61).

Firms’ decisions about expansion also have a spatial element. A firm may start at certain locations and expand geographically. Initial locations of production may be affected by access to technology (close to a university where the innovation was made) and access to intermediate inputs and skilled labor. Location of sales may be affected by relative demand as well as transaction costs and regulations. International expansions through foreign direct investment (FDI) to developing countries are associated frequently with “product cycles” (62). The innovator firm produces the early version of the product in the foreign market and frequently introduces an upgraded, differentiated version of the innovation to the home market; both actions increase the diffusion of the original and upgraded innovation. Vernon (62) illustrated his model with the example of selling older models of washing machines in new less developed markets. Another example is McDonald’s, which after learning and improving the technology in a few pilot outlets, established outlets throughout the United States and later, internationally. The McDonald’s corporation in the United States controls the technology and provision of inputs but mainly relies on franchisees for local management and some investment in facilities.^‖^ McDonald’s emphasizes product consistency over its locations across countries but slightly modifies some of its offerings to fit local tastes (63).

The geographical diffusion of innovations emanating from the home country affects not only the final products available to consumers but also, the market organization and institutions internationally. Globalization over the past several decades, with the associated liberalization of trade and FDI, has led to the spread of market innovations introduced in the United States and western Europe in the 1920s to 1950s, such as supermarkets and fast-food chains, into developing regions in Asia, Latin America, eastern Europe, and Africa (35).

Our analysis is limited, being based mostly on findings in economics and marketing. Further development of our approach should incorporate insight from the rich literature in business and engineering. Beamon (64) reviews the rich literature on design management and analysis of supply chains. It emphasizes operational management issues and design of logistics for multilayer supply chains. The review presents multiple methods of optimization and assessment of supply chain performance, emphasizing methods to incorporating consumer preferences and acceptance. There is growing research on sustainable supply chains, with emphasis on containing pollution and relying on renewable systems (65). The survey by Wong and Ngai (66) critically reviews research on supply chain innovations, which include developments in logistics, organization, marketing, and technologies. Much of this literature is based on experience in developed countries. One of the lessons of our analysis is that with FDI and globalization, supply chains are expanding globally with some lag in their offering, following Vernon’s product cycle model. There is a need for multidisciplinary research on supply chains combining economics, engineering, and business perspectives from a global vantage point.

Government policies that support research directly benefit the ISC but also, lead to the establishment of new PSCs. Financial incentives (penalties on pollution, tax incentives) and regulations that alter the demand for the final product or cost of input affect the performance and evolution of supply chains. For example, carbon pricing can lead to the adoption of greener practices. Investment in infrastructure and education may accelerate the development of supply chains and its use of digital technology. Policies that reduce barriers to trade may result in both globalized supply chains and FDI that will transfer knowledge and capital to developing countries, either expanding existing supply chains or developing new ones.

## Bridging ISC and PSC

There are feedback loops between the ISC and the product PSC. Recall that the ISC fulfills three functions for a new product or technology: 1) discovery or invention, 2) upscaling, and 3) commercialization. The PSC has three functions to implement the ISC-produced innovation: 1) assessment of market potential, 2) supply chain design and implementation, and 3) marketing.

First, there is a feedback loop among the ISC segments. Examples of this are found in agricultural research systems where an initial variety is bred, and then, through testing and initial commercialization attempts, flaws are discovered, forcing the basic research team to adapt. The initial version of high-yield wheat varieties developed in the Green Revolution were tall but top heavy and lodged with wind; the next version was a dwarf variety that was less vunerable to wind (67).

Second, there are ISC–PSC feedback loops. In the implementation and marketing stages of the PSC, the marketing team discovers 1) the need for changes in the design of the initial innovation to better meet consumers’ needs, 2) the need for complementary products to be added to a bundle, and 3) opportunities for product differentiation as part of the product cycle. For example, drip irrigation was introduced originally to save water, but its use was expanded for fertigation and chemigation, leading to the production of complementary chemicals (40). Furthermore, marketers have continuously discovered differentiated needs across segments of users, resulting, for example, in tractors and other farm machinery of various sizes and capabilities (41).

The ISC affects the PSC by introducing new versions of products that utilize new technologies. One example is the movement from vacuum tubes to transistors that radically changed radios in terms of function and capability, eventually leading to significant differentiation. Eventually, the ISC may result in innovations that lead to new product categories with their own PSCs.

The ISCs and PSCs can be inseparable. Developments leading to innovations in ISCs may depend on the capabilities of PSCs. There are also financial links. Government investments in research and education may finance the early phases and basic research of the ISC. Development and upscaling may be financed by venture capitalists or corporations, with some government support. The PSC is more likely to be financed by the private sector.

However, the public sector may support investment in supply chains for innovations with properties of public goods. The ISC and PSC of radical, large-scale innovations with public good properties may be intertwined in the early stages of their commercialization, supported by the public sector. For example, the internet was conceived and initially implemented by the US Defense Advanced Research Projects Agency.

In some cases, the ISC is involved in designing the PSC. For example, the introduction of hydrogen as a fuel requires solving a coordination failure, namely developing supply chains and technologies to refine hydrogen, creating a distribution network, and creating the machinery that consumes hydrogen (68). National laboratories working with universities and companies are establishing road maps for the hydrogen economy,** a basis for the development of policies and commercialization strategies.

The literature on the policy impacts of innovation would benefit from explicitly accounting for both ISC and PSC. The links between research, innovation, and new supply chains suggest that there are benefits from continuous government support of public research and innovation, especially in the early stages of development. It provides a mechanism for how innovations can start new supply chains and lead to the evolution of new industries. Incumbents may use their political clout to reduce government investment or research and slow the evolution of innovation that endangers their market power. Governments tend to underinvest in research and development compared with socially desired levels (70). Incumbents may also abuse their market power, thwart the growth of competitors, and underpay their suppliers. Public sector investment in research, leading to new innovations and products, serves as a mechanism of technological renewal that may mitigate market power

## Implications for Environment, Food, and Natural Resources

The framework presented here can be used to analyze the impacts of and policy responses to climate change as well as other food, environmental, and health challenges. There is a broad literature on the impacts of climate change on smallholders, especially in developing countries, that emphasizes technology adoption and adaptation at the farm level. However, Reardon and Zilberman (71) suggest that it is insufficient to understand the impact on agricultural production in a specific location while neglecting to recognize linkages between farmers and markets. A tsunami that prevents access to a port may endanger food security as much as a drought. Furthermore, international supply chains that recognize vulnerability of certain locations may diversify to reduce reliance on areas with vulnerable linkages, directly affecting the food security and livelihood of small producers. This suggests that enhancing resilience is not only based on improved practices on the farm but also, the design of the supply chain to be more resilient to shocks.

Similarly, the decarbonization of the economy will require continuous research investments as well as the establishment of new industries and conversion of existing sectors to drastically reduce their greenhouse gas emissions. Policy design must account for the industry’s capacity to establish and modify supply chains in addition to the impacts of policy on individual markets. With all their limitations, the US and Brazilian biofuel industries emerged within 15 y because of mandates that assured demand. Through learning by doing, their performance drastically improved, overcoming many of the earlier criticisms (72). However, Clancy and Moschini (73) suggest that financial incentives, like a carbon tax, are preferable to mandates in promoting breakthrough innovation, like second generation biofuels.

The expansion of renewables would not have been possible without early support policies. For example, despite their flaws, subsidies for electric cars and solar energy in the United States and elsewhere have been major contributors to the establishment of these new industries. The development of policies that may lead to the use of hydrogen fuel cells to decarbonize the heavy transportation sector requires understanding the impacts of policies on supply chains.

The literature on food security and rural development has begun to recognize the importance of analyzing food systems and food value chains (74). These food supply chains consist of upstream farmers, midstream processors and wholesalers, and downstream retailers. All segments have responded to innovation (new varieties, cooling and storage technologies, transportation, and information technologies) as well as globalization. This literature suggests that the same processes that led to intensification and urbanization in developed countries are likely to occur in developing countries but at a much faster rate. This literature documents the transformation from traditional supply chains (wet markets and small shops and so on) to modern supply chains, including supermarkets and modern processing facilities. Different transition paths have been detected, and better understanding of food systems would allow for improved research, education, and policy intervention. For example, lack of realization of the transformation of supply chains in developing countries affects policy design and the direction of public research (74). Sexton (75) suggested that analyzing policies affecting the agricultural and natural resources sectors will benefit from modeling capable of addressing emerging innovations, institutional structures, and supply chains in the agricultural and natural resources sectors.

The recent COVID-19 pandemic led to the introduction of various social distancing policies. Assessing the impact of these policies requires an understanding of the workings of food supply chains. Reardon et al. (76) argue that mobility restrictions regulations will mostly affect traditional and transitional labor-intensive food supply chains and small firms. This may lead to the consolidation of the food supply chain in developing countries. Furthermore, food retailers (e.g., supermarkets and restaurants) have pivoted from selling directly to consumers to utilizing e-commerce facilitated by “delivery intermediaries.” Laborers in the traditional sector are likely to be negatively affected without a sufficient safety net. Agricultural production may not be affected directly by mobility restrictions and social distancing, but constraints on migration and breakdowns of supply chains may adversely affect farmers. Policy analysis based on welfare economics (77) should consider the impacts of shocks on existing supply chains, possible mechanisms of adaptation, and the resulting impacts on consumers and the economy.

## Conclusions

This paper integrates several bodies of literature to develop a framework to analyze the transition from new ideas to final products. This transition is done through two symbiotic supply chains, the ISC and the PSC. These supply chains are affected by policies including support for research and education, intellectual property rights protection, antitrust policies to strengthen competition, low barriers to trade and light regulation, and well-functioning capital markets. The notions of ISP and PSC apply globally, but there are significant differences among locations and sections, reflecting different stages of development and policies. Furthermore, globalization has led to diffusion of institutions and products globally.

There is a vast need for more empirical work on supply chains. It may require better documenting and understanding of the behavior of large firms; the evolution of linkages between them; and especially in developing countries, the behavior of small and medium enterprises, especially in the midstream segments of supply chains. Analysis of these patterns can be challenging, as data are rarely well organized or easily available. Analysis of supply chains' evolution may require the development or application of tools to analyze narratives and case studies to supplement traditional data sources used by economists. It will also need to understand the choices of engineers who design supply chains as well as policy makers who provide funding for infrastructure and create regulations that affect supply chains. Economics has evolved to be able to address these challenges. The basic premise of economics is that agents pursue their self-interest subject to constraints of ability and institutions. In the twentieth century, behavioral economics and political economy recognized that the traditional classical economic model has its limitations in analyzing individual behavior and social choice. In the twenty-first century, economic frameworks need to evolve to become multidisciplinary and incorporate political, technological, and engineering considerations and tools.

## Data Availability

There are no data underlying this work.

## Footnotes

^*^The expectations are rational in the sense that they are the best given available information. The extent to which they tend to be fulfilled on average (6) needs to be tested empirically.

^†^In this case, $X_{I}=0,X=X_{E,\;}$ and MREV(X)= MPROC(X)+MINFC (X),  $\text{where}\;\mathrm{MREV}\left(X\right)=\frac{\mathrm{dREV}}{dX}$ and the other marginal relationships are determined accordingly.

^‡^In this case, $\mathrm{MINFC}(X_{I})=\;\mathrm{MEXPC}(X_{E\;})$ and $\mathrm{MREV}\left(X_{I}+X_{E,\;}\right)=\;\mathrm{MPROC}\left(X_{I}+X_{E\;}\right)+\mathrm{MINFC}\;\left(X_{I}\right)$.

^§^In this case, $X_{E\;}=0,X_{I}=X$, and MREV(X)= MPROC(X)+MEXPC(X). That is, the marginal purchasing cost MEXPC(X) is higher than the marginal cost of external inputs since the marginal purchasing cost takes into account that increased purchases tend to increase the per unit price of the input.

^¶^$\mathrm{VMB}I_{t}=\overset{T}{\underset{j=t+1}{\sum }}\frac{{\left(1-\delta \right)}^{t-j}}{{\left(1+r\right)}^{t-j+1}}\left(\mathrm{MREV}K_{j}\left(X_{j},K_{j}\right)-\mathrm{MPROC}K_{j}(X_{j},K_{j})\right)=1;\;\;\mathrm{MREV}K_{j}=\frac{\partial \mathrm{REV}}{\partial K}$; and $\mathrm{MPROC}K_{j}=\frac{\partial \mathrm{PROC}}{\partial K}$.

^#^In more oligopolistic markets, technological improvement may increase overall supply and lead to increased adoption, but production of individual differentiated products may not increase because of price differentiation.

^‖^McDonald’s actually owns all the real estate where some of the franchises are located. In other cases, the innovator may own the technology and control the supply of products, but franchises may own or rent much of the real estate.

^**^Information is available in ref. 69.
